# Supplementary material for: Maternal glucocorticoid levels during incubation predict breeding success, but not reproductive investment, in a free-ranging bird
Source: Biol Open. 2020 Oct 19;9(10):bio045898. doi: 10.1242/bio.045898 (PMC7595688; doi:10.1242/bio.045898)
Supplement: Supplementary information [file biolopen-9-045898-s1.pdf]

Table S1. Global statistical models describing variation in female tree swallows in each treatment group (Sham, CORT, and Control). Statistical and mean values (raw mean ± SE) are from results of linear models. Females allocated to each treatment did not differ statistically from each other.

Response variable	Sham	CORT	Control	Fixed effects	β	SE	df	t	P	R^2
**Pre-laying body mass (g)**	21.3±0.43 N=19	22.1±0.46 N=13	---	Intercept	23.093	0.683	1, 27	33.811	<0.001	0.101
				Treatment (Sham)	-0.783	0.593	1, 27	-1.321	0.198	
				Age (ASY)	-0.411	0.665	1, 27	-0.618	0.542	
				Site (Nature Area)	0.661	0.585	1, 27	1.129	0.269	
				Year	-0.903	0.572	1, 27	-1.578	0.126	
**Flattened wing length (mm)**	114.9±0.66 N=20	115.7±0.72 N=14	116.6±0.71 N=15	Intercept	116.806	0.963	1, 43	121.327	<0.001	0.169
				Treatment (CORT)	-0.980	1.042	1, 43	-0.940	0.352	
				Treatment (Sham)	-1.699	1.022	1, 43	-1.662	0.104	
				Age (ASY)	2.022	0.870	1, 43	2.323	**0.025**	
				Site (Nature Area)	0.020	0.781	1, 43	0.026	0.979	
				Year	-2.334	0.762	1, 43	-3.062	**0.004**	
**Clutch initiation date (Julian day)**	142.9±1.19 N=20	142.9±1.32 N=14	141.3±1.13 N=20	Intercept	144.917	1.704	1, 48	85.059	<0.001	0.339
				Treatment (CORT)	1.581	1.768	1, 48	0.894	0.376	
				Treatment (Sham)	1.583	1.697	1, 48	0.933	0.356	
				Age (ASY)	-7.027	1.480	1, 48	-4.747	**<0.001**	
				Site (Nature Area)	2.679	1.387	1, 48	1.931	**0.059**	
				Year	2.458	1.361	1, 48	1.806	0.077	

Control birds did not have a pre-laying body mass measured because they were not captured until incubation
Significant main effects are bolded.
